# Effects of CsSn_*x*_Pb_1−*x*_I_3_ Quantum Dots as Interfacial Layer on Photovoltaic Performance of Carbon-Based Perovskite Solar Cells

**DOI:** 10.1186/s11671-021-03533-y

**Published:** 2021-04-29

**Authors:** Chi Zhang, Zhiyuan He, Xuanhui Luo, Rangwei Meng, Mengwei Chen, Haifei Lu, Yingping Yang

**Affiliations:** Department of Physics, School of Science, Wuhan University of Technology, Wuhan, 430070 People’s Republic of China

**Keywords:** Tin-doped perovskite quantum dots, Photovoltaic performance, Carbon-based perovskite solar cells

## Abstract

In this work, inorganic tin-doped perovskite quantum dots (PQDs) are incorporated into carbon-based perovskite solar cells (PSCs) to improve their photovoltaic performance. On the one hand, by controlling the content of Sn^2+^ doping, the energy level of the tin-doped PQDs can be adjusted, to realize optimized band alignment and enhanced separation of photogenerated electron–hole pairs. On the other hand, the incorporation of tin-doped PQDs provided with a relatively high acceptor concentration due to the self-p-type doping effect is able to reduce the width of the depletion region near the back surface of the perovskite, thereby enhancing the hole extraction. Particularly, after the addition of CsSn_0.2_Pb_0.8_I_3_ quantum dots (QDs), improvement of the power conversion efficiency (PCE) from 12.80 to 14.22% can be obtained, in comparison with the pristine device. Moreover, the experimental results are analyzed through the simulation of the one-dimensional perovskite/tin-doped PQDs heterojunction.

**Supplementary Information:**

The online version contains supplementary material available at 10.1186/s11671-021-03533-y.

## Introduction

In past few years, perovskite materials have been widely applied in solar cells due to their excellent electrical and optical properties, such as suitable bandgap width, large light absorption coefficient and good defect tolerance [1–6]. Interface engineering, as a strategy to modify the interface characteristics of thin film devices, has become one of the approaches to improve the performance of perovskite solar cells (PSCs) [7, 8]. Recently, lead-based halide perovskite quantum dots (PQDs) in the form of APbX_3_ (A = CH_3_NH_3_^+^ (MA^+^), Cs^+^; X = Cl^−^, Br^−^, I^−^) are often used as interfacial layers or additives for optimized band alignment thanks to their adjustable band structures [9–15]. The combination of perovskite absorbers and PQDs is regarded as an effective method for enhanced charge extraction and improved PSC properties.

It is worth noting that most of the relevant researches are based on PSCs with hole-transporting layers (HTLs). In recent years, carbon-based HTL-free PSCs with simple preparation processes and low costs have been given much attention [16–18]. Similarly, PQDs can also be used in this PSC structure. However, some other requirements besides band alignment should be taken into consideration. First, the lattice structures of lead-based PQDs are not very stable due to Pb^2+^ with a large ionic radius reducing the tolerance factor. Therefore, lead-reduced PQDs are promising candidates. Second, because of the lack of HTLs, the hole transport performance is bound to be weakened. Consequently, the added PQDs are required to supply extra free holes, so that photogenerated holes can be smoothly transferred from the perovskite layer to the carbon electrode.

The ion exchange method using metal cations with smaller ionic radii (such as Cu^2+^, Zn^2+^, Sn^2+^, Cd^2+^) to partially replace Pb^2+^ has been proven to improve the lattice stability of PQDs [19–21]. Among these metal cations, Sn^2+^ is easily to oxidize to Sn^4+^, which can introduce self-p-type doping effects to enhance hole transfer [22–24]. Particularly, Liu et al. synthesized CsSn_0.6_Pb_0.4_I_3_ quantum dots (QDs) featuring a hole mobility of 40.12 cm^2^ V^−1^ s^−1^ and good stability in the ambient air [25]. Xu and co-workers incorporated CsSnBr_3−*x*_I_*x*_ QDs between the CsPbBr_3_ perovskite and the carbon electrode to promote charge extraction [26]. Very recently, Duan et al. found that MAPbI_3_/CsSnI_3_ heterojunction as the light-harvester in the carbon-based HTL-free PSC could facilitate the hole transfer [27]. Inspired by these above, we propose that tin-doped PQDs with appropriate energy levels and self-p-type doping effects are able to function like HTLs to modify the injection and transport characteristics of holes.

In this work, tin-doped PQDs in the form of CsSn_*x*_Pb_1−*x*_I_3_ were incorporated between the MAPbI_3_ perovskite and the carbon electrode to achieve optimized band alignment and improved hole transfer. An increment in power conversion efficiency (PCE) of 11.09%, from 12.80 to 14.22%, could be obtained after the addition of CsSn_0.2_Pb_0.8_I_3_ QDs.

## Methods

### Materials

Tin iodide (SnI_2_; 99.99%) was bought from Youxuan Technology (China). Cesium carbonate (Cs_2_CO_3_; 99%), 1-octadecene (ODE; > 90%), oleic acid (OA; 99%), oleylamine (OAM; 80–90%), methyl acetate (MeOAc; 98%) and trioctylphosphine (TOP; 90%) were purchased from Macklin (China). Lead iodide (PbI_2_; 99.99%) and methylammonium iodide (MAI; 99.5%) were obtained from Xi’an p-OLED (China). Titanium diisopropoxide bis (acetylacetonate; 75%), dimethylsulfoxide (DMSO; 99.9%) and *N*,*N*-dimethylformamide (DMF; 99.8%) were purchased from Sigma-Aldrich (US). The TiO_2_ paste (30NR-D) and the low-temperature carbon electrode paste were obtained from Shanghai MaterWin New Materials (China).

### Synthesis and Purification of Tin-Doped PQDs

We adopted a simple mixed-heating procedure to synthesize tin-doped PQDs. Briefly, Cs_2_CO_3_, SnI_2_ and PbI_2_ with a specific molar ratio (CsSn_0.1_Pb_0.9_I_3_ QDs: 0.037 mmol Cs_2_CO_3_, 0.2 mmol PbI_2_, 0.15 mmol SnI_2_; CsSn_0.2_Pb_0.8_I_3_ QDs: 0.037 mmol Cs_2_CO_3_, 0.2 mmol PbI_2_, 0.2 mmol SnI_2_; CsSn_0.3_Pb_0.7_I_3_ QDs: 0.037 mmol Cs_2_CO_3_, 0.2 mmol PbI_2_, 0.25 mmol SnI_2_) were mixed with 10 mL of ODE, 0.5 mL of OA, 0.5 mL of OAM and 0.5 mL of TOP in a 50-mL three-neck flask. OA, OAM and TOP were utilized to limit the particle size and to passivate the surface defects of tin-doped PQDs. Then, the mixture was stirred and heated at 100 °C for 30 min under nitrogen atmosphere to obtain a red solution, including nano-sized and micron-sized tin-doped perovskites. To extract and purify tin-doped PQDs, 10 mL of MeOAc was added into the red solution, followed by centrifuging at 7000 rpm for 5 min. The supernatant was discarded, and the brown black precipitate was dispersed in 5 mL of hexane. Finally, the brown black solution was centrifuged at 3000 rpm for 5 min, and the red supernatant contained only the tin-doped PQDs.

### Device Fabrication

Fluorine-doped SnO_2_ (FTO) glasses were washed with water, acetone, isopropanol and ethanol in sequence for 30 min each in an ultrasonic cleaner. After that, the FTO glasses were treated by ultraviolet (UV) for 20 min to remove residual organic solvents. The compact TiO_2_ (c-TiO_2_) layer was fabricated on the FTO layer by spin-coating a solution of acetylacetonate (0.1 mL) diluted in ethanol (1.9 mL) with the speed of 4000 rpm for 30 s. Then, the glasses were annealed at 150 °C for 5 min and at 500 °C for 30 min. Subsequently, the mesoporous TiO_2_ (m-TiO_2_) layer was obtained by spin-coating a solution of TiO_2_ paste diluted in ethanol onto the c-TiO_2_ layer at 3500 rpm for 20 s and annealed at 500 °C for 30 min. The annealing process at 500 °C is to obtain TiO_2_ layers with improved electron transport performance. Next, to prepare the MAPbI_3_ precursor solution, PbI_2_ (0.5 mmol) and MAI (0.5 mmol) were mixed with DMF (300 mg) and DMSO (39 mg). Afterward, the MAPbI_3_ layer was fabricated by spin-coating the MAPbI_3_ precursor solution (35 μL) onto the m-TiO_2_ layer, with the speed of 1000 rpm for 10 s and 4000 rpm for 20 s, followed by heating at 100 °C for 10 min. After that, tin-doped PQDs dispersed in toluene (10 mg mL^−1^) were spin-coated onto the perovskite layer at 4000 rpm for 30 s and annealed at 90 °C for 5 min to remove the residual toluene. Finally, the carbon electrode paste was screen-printed on the device and annealed at 100 °C for 10 min.

### Characterization

Transmission electron microscope (TEM) images, selected-area electron diffraction (SAED) views and energy-dispersive X-ray spectroscopy (EDS) analyses of tin-doped PQDs were obtained by a field emission high-resolution transmission electron microscope (JEM-2100F, JEOL, Japan) at an accelerating voltage of 200 kV. The valence band (VB) edges of different materials were acquired from an X-ray photoelectron spectrometer (ESCALAB 250Xi, Thermo Fisher Scientific, USA). Absorption and steady-state photoluminescence (PL) characteristics were collected via an UV–visible spectrophotometer (UV-3600, Shimadzu, Japan) and a fluorescence spectrometer (RF-6000, Shimadzu, Japan), respectively. The cross-sectional image of the PSC and the surface morphologies of perovskite films were obtained by a scanning electron microscope (Zeiss Ultra Plus, Zeiss, Germany). Curves of photocurrent density versus on voltage (*J*–*V*) were measured by a sourcemeter (2400, Keithley, USA) with a sunlight simulator (Oriel Sol3A, Newport, US), under AM 1.5G simulated illumination (100 mW cm^−2^). Monochromatic incident photon-to-electron conversion efficiency (IPCE) spectra and electrochemical impedance spectroscopies (EIS) were obtained from an electrochemical workstation (Zahner, Kronach, Germany). Finally, X-ray diffraction (XRD) patterns of perovskite films and PQDs were acquired from an X-ray diffractometer (Empyrean, PANalytical, Netherlands).

## Results and Discussion

**Fig. 1:**
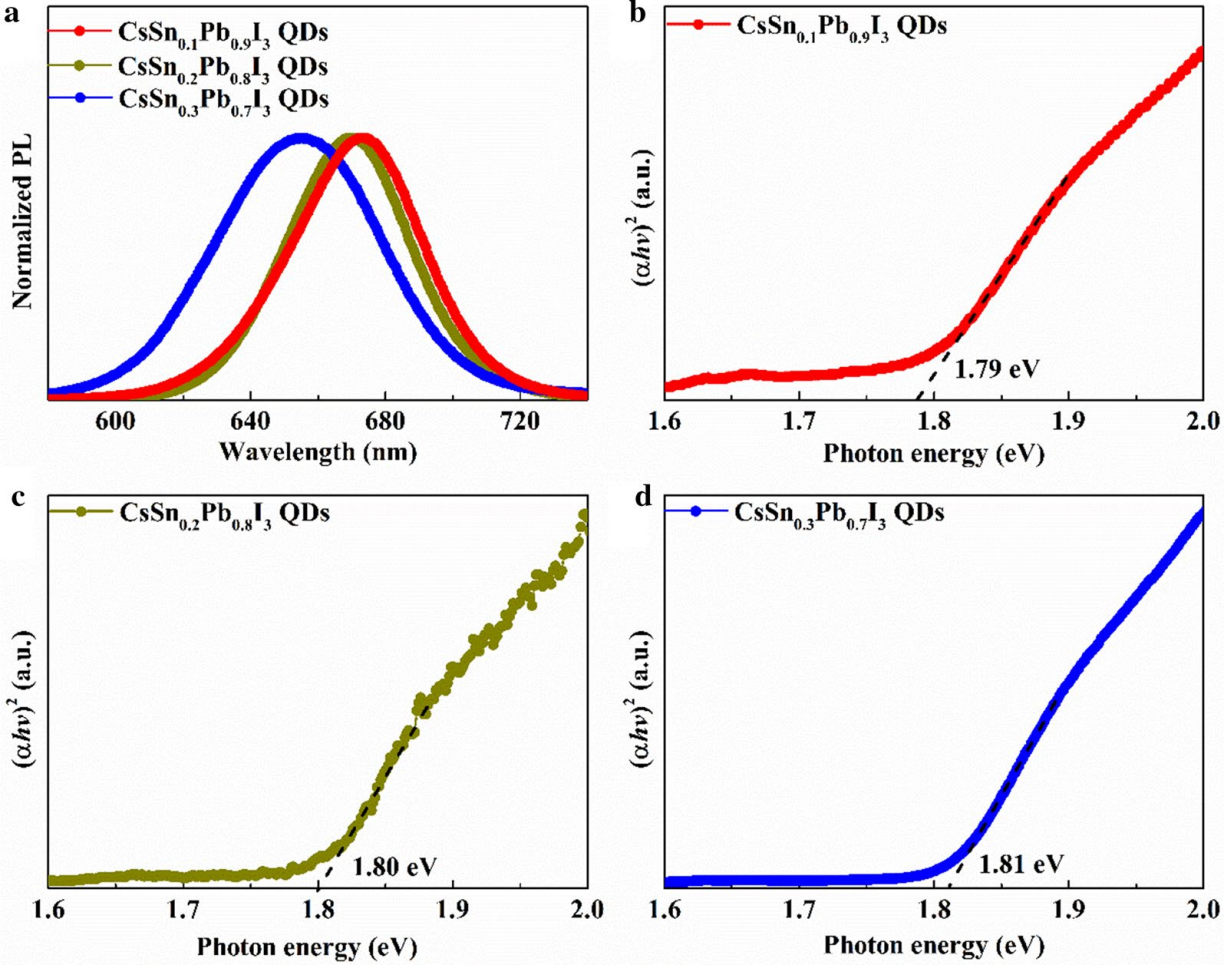
**a** Normalized PL spectra and **b-d** Tauc plots of different tin-doped PQDs

Three kinds of tin-doped PQDs were studied in this work, including CsSn_0.1_Pb_0.9_I_3_ QDs, CsSn_0.2_Pb_0.8_I_3_ QDs and CsSn_0.3_Pb_0.7_I_3_ QDs. The actual atomic ratios of Sn/(Sn + Pb) in these PQDs were estimated to be 13.03%, 22.12% and 32.57%, respectively (shown in Additional file 1: Fig. S1 and Tables S1–S3). As shown in Fig. 1, blue-shifts of the steady-state PL peak (673 nm, 669 nm and 656 nm in turn) and the edge of Tauc plot (1.79 eV, 1.80 eV and 1.81 eV in turn) were observed with the increase of Sn doping. For many bulk perovskite materials in the form of ABX_3_ (A = Cs, MA, FA; B = Sn_*x*_Pb_1−*x*_; X = Cl, Br, I), the bandgap often exhibits a downward trend with the increase of the *x* value. That is because the bandgap is determined by the electronegativity of the B-site atom (Pb^2+^: *χ* = 1.6; Sn^2+^: *χ* = 1.7). However, when it comes to nanocrystals with quantum confinement, the impact of unit cell volume on bandgap matters more. The perovskite bandgap is known to increase with the decrease of the unit cell volume [19]. Therefore, more Sn^2+^ substitution would further intensify the lattice contraction, which led to the augment of bandgap width, consistent with the reported research [28]. Meanwhile, the larger electronegativity of Sn atom might be the reason why the bandgap did not increase significantly.

**Fig. 2:**
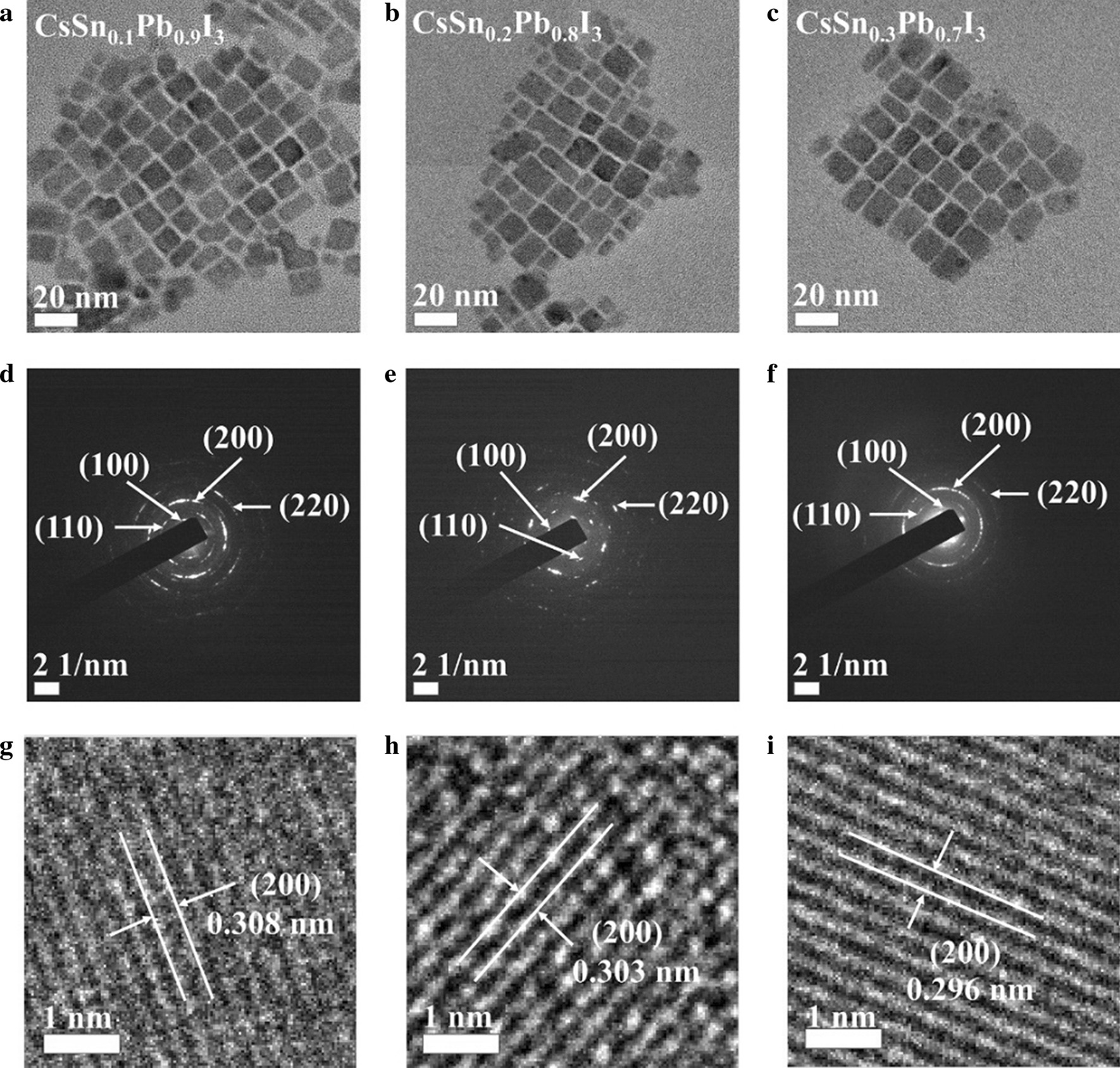
**a**–**c** TEM images, **d**–**f** SAED patterns and **g**–**i** enlarged TEM images of CsSn_0.1_Pb_0.9_I_3_ QDs, CsSn_0.2_Pb_0.8_I_3_ QDs and CsSn_0.3_Pb_0.7_I_3_ QDs

The TEM images of these tin-doped PQDs are exhibited in Fig. 2a–c. These tin-doped PQDs were all square, consistent with the theoretical lattice structure of cubic phase. Besides, the average size of each of these three PQDs was about 15 nm, and there was no significant difference. That was because the size was mainly determined by the reaction temperature, which was kept at 100 °C for all the PQDs. Besides, SAED measurements are shown in Fig. 2d–f. By comparing the interplanar spacing values of different diffraction rings with corresponding standard values (CsPbI_3_ in cubic phase, ICSD, 181288), some crystal planes including (100), (110), (200) and (220) could be identified, which also indicated that these tin-doped PQDs were mostly composed of cubic nanocrystals (NCs) [20]. Moreover, enlarged TEM images shown in Fig. 2g–i are utilized to probe the crystal plane characteristics. The interplanar distances of (200) plane of these tin-doped PQDs were determined to be 0.308 nm, 0.303 nm and 0.296 nm, in turn, which demonstrated that increasing substitution of Pb^2+^ by Sn^2+^ led to the lattice shrinkage, in accordance with their optical characteristics mentioned above.

**Fig. 3:**
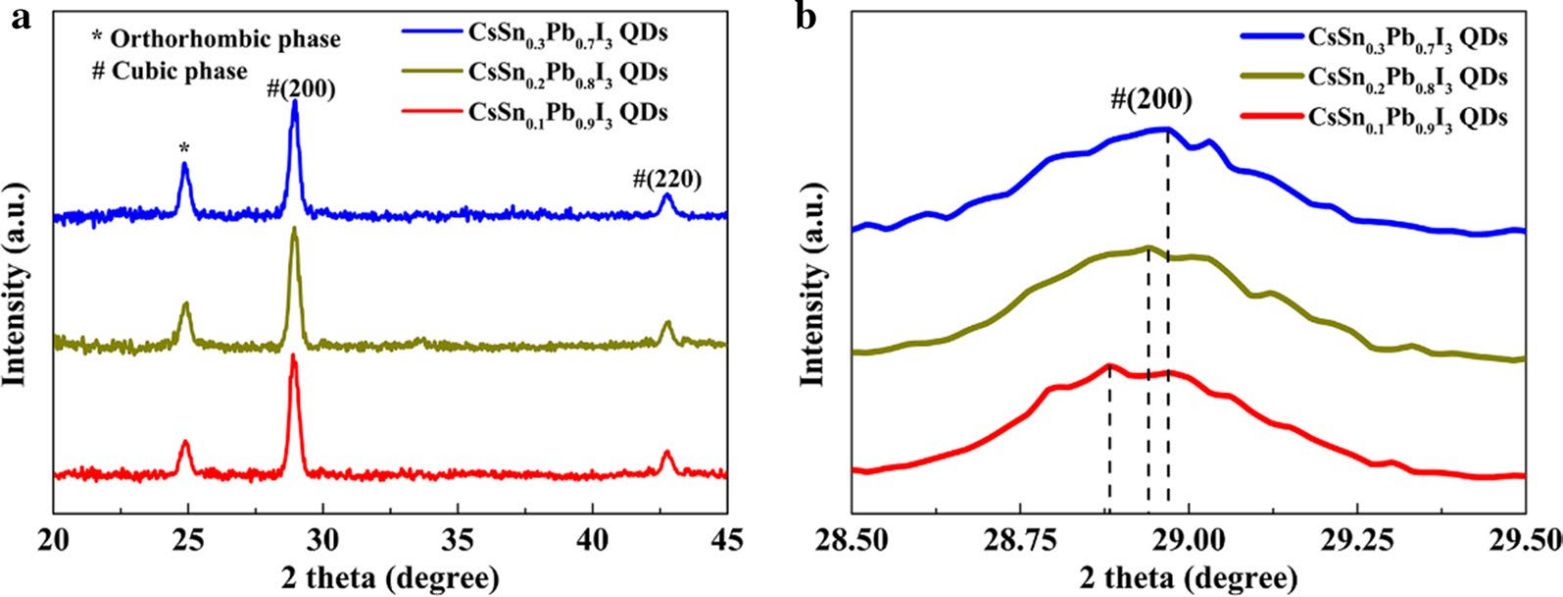
**a** Normalized XRD patterns of different tin-doped PQDs. **b** Enlarged XRD patterns for (200) planes

To further study the lattice structures of these tin-doped PQDs, we performed normalized XRD measurements, shown in Fig. 3. According to the standard XRD data of CsPbI_3_ in orthorhombic and cubic forms [20, 29], the diffraction peaks associated with orthorhombic and cubic phases were marked with “*” and “#,” respectively. As the amount of Sn doping in PQDs increased, the diffraction angle of the peak corresponding to (200) plane slightly increased, implying that the interplanar distance of (200) plane was reduced, in line with the analysis above. Meanwhile, the intensity of the diffraction peak representing orthorhombic phase showed an increasing trend, which indicated that the phase transition process in the PQDs increased. This might be because the increase in the amount of Sn doping would intensify the oxidation reaction of the PQDs in the air, resulting in more Sn vacancies, which may make Pb refill these vacancies to form an unstable perovskite structure.

**Fig. 4:**
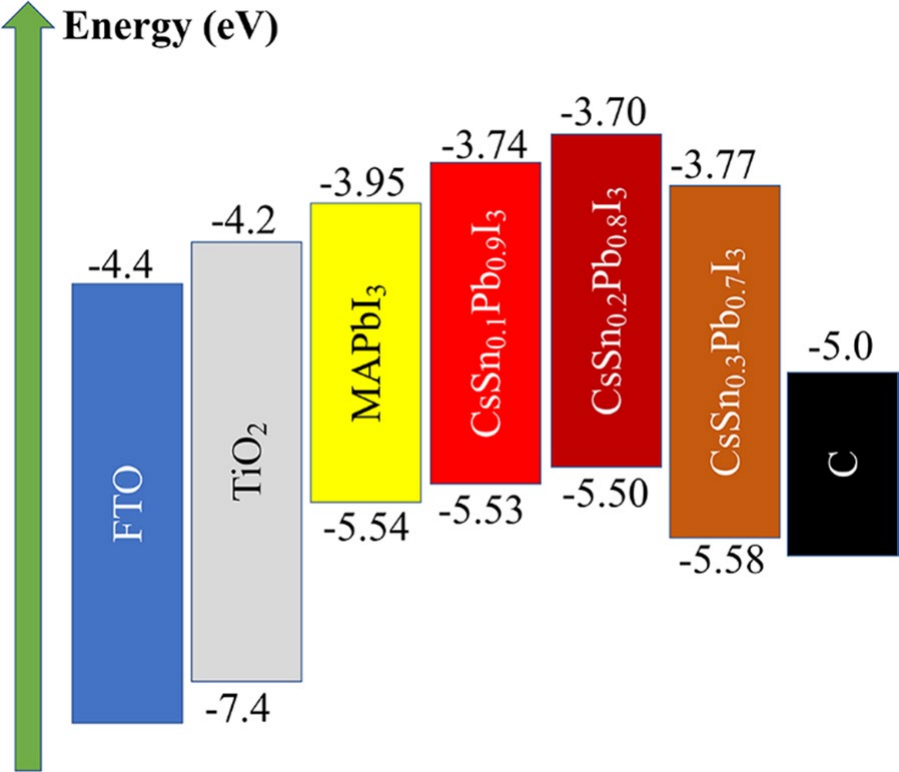
Band structures of different materials in PSCs

Optimized band alignment is crucial for enhancing the extraction of photogenerated carriers and suppressing non-radiative recombination [30–33]. Figure 4 shows the band structures of various materials including FTO, TiO_2_, MAPbI_3_, CsSn_0.1_Pb_0.9_I_3_ QDs, CsSn_0.2_Pb_0.8_I_3_ QDs, CsSn_0.3_Pb_0.7_I_3_ QDs and carbon. Corresponding UPS data and Tauc plots are shown in Additional file 1: Fig. S2. It is clear that the valence band (VB) edge of CsSn_0.1_Pb_0.9_I_3_ QDs (− 5.53 eV) or CsSn_0.2_Pb_0.8_I_3_ QDs (− 5.50 eV) was higher than that of MAPbI_3_ (− 5.54 eV), satisfying the band alignment requirement. It was able to eliminate the large Schottky barrier formed by the MAPbI_3_/carbon junction, thus enhancing the hole extraction ability (discussed later) [31]. Furthermore, the higher conduction band (CB) edges of these tin-doped PQDs were expected to hinder the flow of electrons from MAPbI_3_ to the carbon electrode. However, the VB edge of CsSn_0.3_Pb_0.7_I_3_ QDs (− 5.58 eV) was lower than that of MAPbI_3_, which would block the hole injection, leading to more charge recombination at the interface between MAPbI_3_ and the PQDs.

Moreover, the VB edge originates from the interactions between Pb (6s) and I (5p) orbitals, which are also determined by the Sn doping amount. On the one hand, the substitution of Pb^2+^ by Sn^2+^ will shrink the lattice structure, leading to shorter Pb–I bonds and stronger interactions between Pb and I orbitals, so that the VB tends to shift to a higher energy position [19]. On the other hand, more lattice distortions (transformation from cubic NCs to orthorhombic NCs) will be introduced into the PQDs with excessive Sn^2+^ substitution, resulting in expanded volume of [PbI_6_] octahedra and weaker Pb–I interactions, thus moving the VB to a lower energy position [21]. As a result, the VB edge does not vary linearly with the Sn doping of the PQDs. A reasonable Sn doping content is the key to obtaining an appropriate band structure.

Unlike ordinary lead-based PQDs, tin-doped PQDs will partially undergo oxidation in air due to the presence of Sn^2+^, described by

1 $$2{\text{CsSn}}_{x} {\text{Pb}}_{{{1} - x}} {\text{I}}_{3} + x{\text{O}}_{{2}} \to x{\text{Cs}}_{{2}} {\text{SnI}}_{{6}} + {(2} - {2}x{\text{)CsPbI}}_{{3}} + x{\text{SnO}}_{{2}}.$$

CsSn_*x*_Pb_1−*x*_I_3_ can be assumed to be the combination of CsSnI_3_ and CsPbI_3_ with a certain molar ratio. Among the compounds, only CsSnI_3_ participates in the oxidation reaction. Then, this process can be simplified to

2 $$2{\text{CsSnI}}_{3} + {\text{O}}_{2} \to {\text{Cs}}_{2} {\text{SnI}}_{6} + {\text{SnO}}_{2}.$$

In reaction (2), the transformation from CsSnI_3_ to Cs_2_SnI_6_ is regarded as breaking the connections between [SnI_6_] octahedra. The reason is that CsSnI_3_ is formed by corner sharing [SnI_6_] octahedra, while Cs_2_SnI_6_ is made up of isolated [SnI_6_] octahedra [22]. Therefore, these half of Sn atoms do not leave the perovskite lattice. However, the other half of the Sn atoms are oxidized to SnO_2_, leaving a lot of Sn vacancies in the lattice, which will accept electrons (or supply holes) and act as p-type dopants. It can be described by Eq. (3) as follows:

3 $${\text{Sn}}^{2 + } + {\text{O}}_{2} \to {\text{SnO}}_{2} + 2{\text{h}}^{ + }.$$

That is the reason for the self-p-type doping effects of tin-doped PQDs. Accordingly, under the premise that the lattice structure of tin-doped PQDs can be stabilized, the acceptor concentration of the PQDs will increase with the Sn doping amount.

**Fig. 5:**
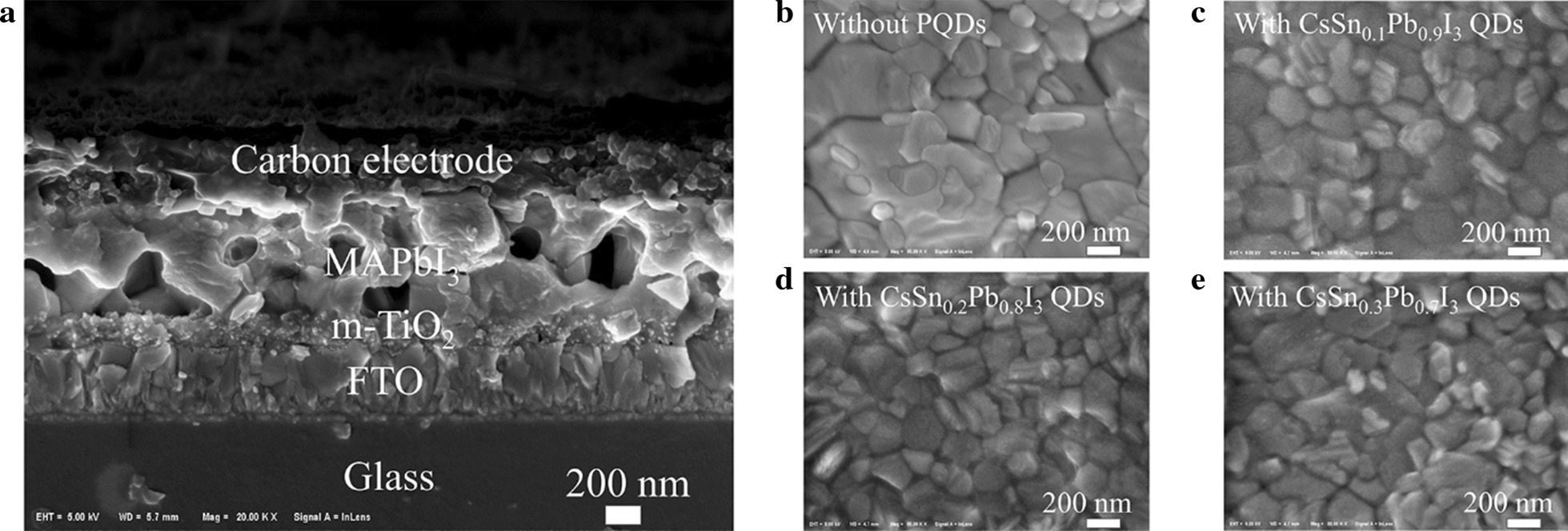
**a** Cross-sectional image of the PSC. **b**–**e** Perovskite films without and with PQDs

**Fig. 6:**
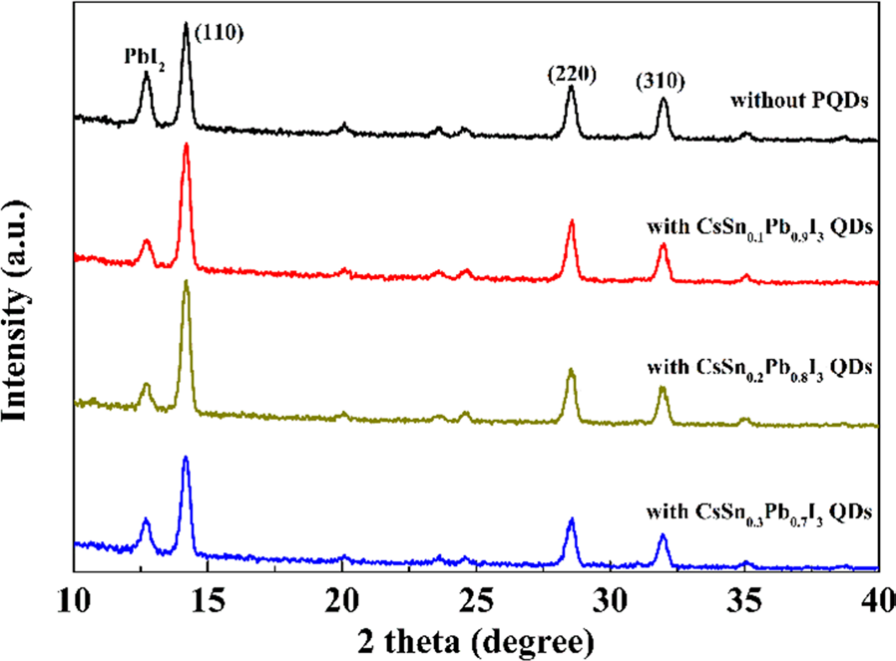
GIXRD patterns of different perovskite films

The cross-sectional image of the PSC is shown in Fig. 5a. The widths of FTO layer, m-TiO_2_ layer and MAPbI_3_ layer were about 400 nm, 200 nm and 800 nm, respectively. Because of the low concentration of the PQD solution (10 mg mL^−1^), it was hard to observe a PQD layer that could be distinguished from the underlying MAPbI_3_ film. To prove the existence of PQDs on MAPbI_3_, we performed XPS measurement on the film with the structure of FTO/c-TiO_2_/m-TiO_2_/MAPbI_3_/PQDs. The XPS results are shown in Additional file 1: Fig. S3. The elements including Cs, I, Sn and Pb were all detected, demonstrating that there was a PQD layer on the perovskite film. Besides, as shown in Fig. 5b–e, there were many small-sized white PbI_2_ particles on the original perovskite film, caused by the partial decomposition of the perovskite in the air. After adding tin-doped PQDs, the number of white particles decreased, and the perovskite films exhibited slightly better grain uniformity and compactness than the pristine sample. However, the morphology difference between the various perovskite films was still not obvious. In order to further distinguish their surface characteristics, we performed grazing incidence XRD (GIXRD) patterns of perovskite films with different tin-doped PQDs, exhibited in Fig. 6. The diffraction peak at about 12.7° is associated with PbI_2_ [34]. After the modification of tin-doped PQDs, the diffraction intensity ratio of PbI_2_:(110) plane was decreased, suggesting that the decomposition process of the perovskite film was suppressed.

**Fig. 7:**
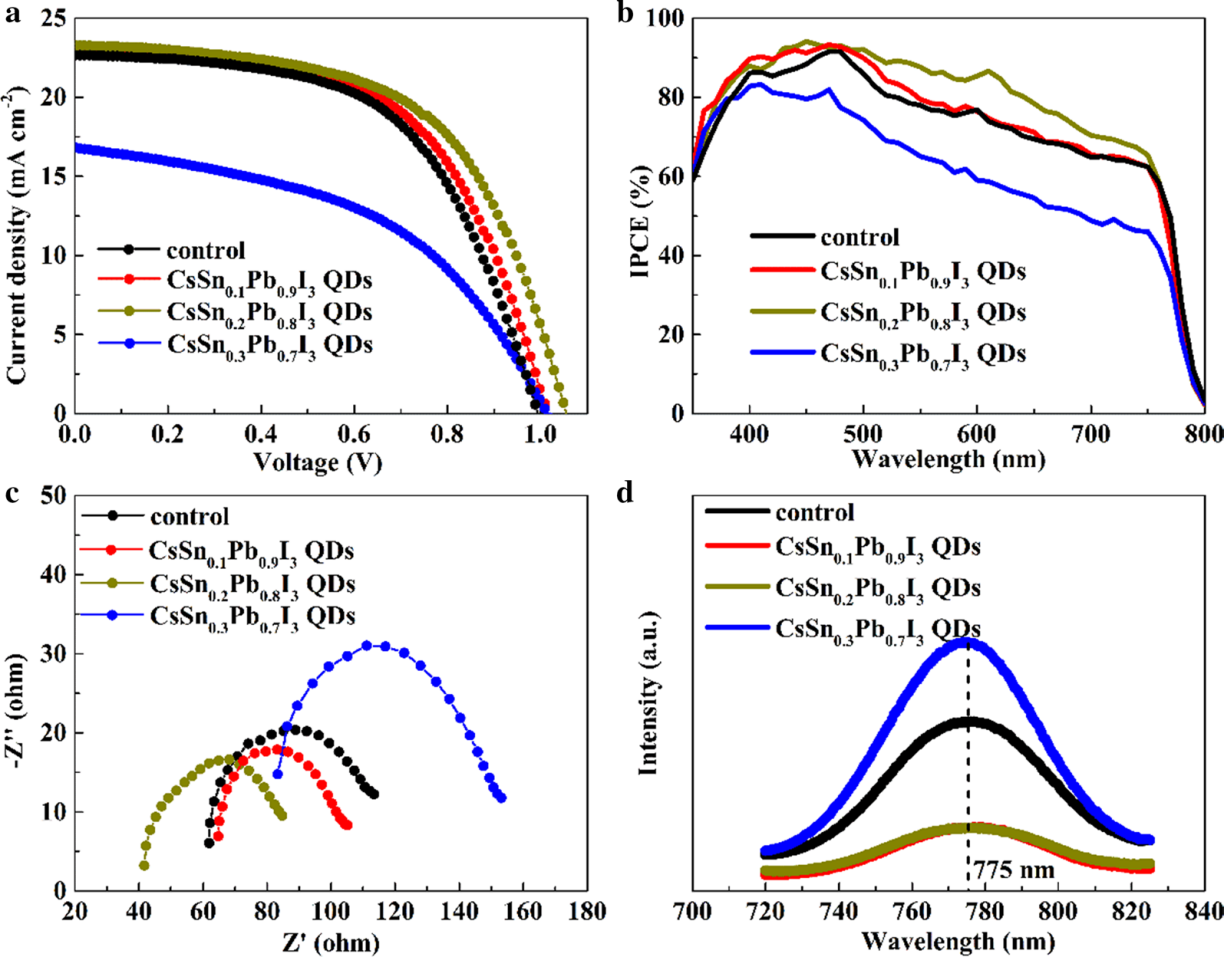
**a** *J*–*V* curves, **b** IPCE spectra and **c** EIS measurements of different PSCs. **d** PL spectra of perovskite films with and without tin-doped PQDs

The curves of photocurrent density versus on voltage (*J–V*) of different PSCs are displayed in Fig. 7a, and photovoltaic parameters including short-circuit current density (*J*_sc_), open-circuit voltage (*V*_oc_), fill factor (FF) and PCE are shown in Table 1. The values of *J*_sc_, *V*_oc_, FF and PCE of the PSC without modification by tin-doped PQDs were 22.69 mA cm^−2^, 0.99 V, 56.78% and 12.80%, respectively. For the CsSn_0.1_Pb_0.9_I_3_ QDs-added PSC, various parameters were improved. However, the improvement was not optimal, which might ascribe to the relatively low Sn doping of the PQDs. In contrast, with the incorporation of CsSn_0.2_Pb_0.8_I_3_ QDs, a *J*_sc_ of 23.30 mA cm^−2^, a *V*_oc_ of 1.05 V, a FF of 57.90% and a PCE of 14.22% could be obtained. The significant increase in each parameter indicated the reduction of non-radiative recombination and the effective extraction of photogenerated holes. Besides, as shown in Additional file 1: Fig. S4, PCE values for 90% of CsSn_0.2_Pb_0.8_I_3_ QDs-added PSCs surpassed 13%, showing good repeatability. For the PSC modified by CsSn_0.3_Pb_0.7_I_3_ QDs, the values of *J*_sc_ and FF seriously dropped to 16.82 mA cm^−2^ and 47.40%, respectively. The lower VB edge of the PQDs would hinder the hole transfer from the MAPbI_3_ film to the carbon electrode. Furthermore, when the Sn content of PQDs was too high, more Sn vacancies would be introduced, resulting in more phase transformation products with large bandgap widths [29, 35–37], thus seriously impeding the transport process of photogenerated carriers.

**Table 1:** Photovoltaic parameters of different PSCs

Device	*V*_oc_ (V)	*J*_sc_ (mA cm^−2^)	FF (%)	PCE (%)	*R*_CT_ (Ω)	*C*_T_ (nF)
Control	0.99	22.69	56.78	12.80	56.5	35.5
With CsSn_0.1_Pb_0.9_I_3_ QDs	1.01	22.85	57.81	13.38	47.0	32.9
With CsSn_0.2_Pb_0.8_I_3_ QDs	1.05	23.30	57.90	14.22	43.0	44.7
With CsSn_0.3_Pb_0.7_I_3_ QDs	1.01	16.82	47.40	8.07	81.4	16.3

As described in Fig. 7b, the IPCE spectra in a wavelength range from 350 to 800 nm increased in the order of CsSn_0.3_Pb_0.7_I_3_ QDs-added device < control device < CsSn_0.1_Pb_0.9_I_3_ QDs-added device < CsSn_0.2_Pb_0.8_I_3_ QDs-added device, in agreement with the corresponding trend of *J*_sc_ acquired from the *J*–*V* curves. It is clear that the difference of these IPCE curves was mainly reflected in the wavelength range from 550 to 800 nm. Tin-doped PQDs added onto the perovskite film would significantly affect the built-in electric field near the back surface of the perovskite (analyzed in detail later). At the same time, the long-wavelength photons were mainly absorbed by the perovskite near the back surface due to their low energy. When these photons were transformed to carriers, their transport properties would be more easily changed by the above-mentioned built-in electric field than those carriers converted from short-wavelength photons.

In addition, EIS measurements, in a frequency range from 4 to 0.2 MHz at a bias of 0.8 V under simulated AM 1.5G radiation, were performed to analyze the charge transport resistance (*R*_CT_) and the barrier capacitance (*C*_T_) near the carbon electrode, described in Fig. 7c. Corresponding EIS parameters are also shown in Table 1. With the addition of CsSn_0.2_Pb_0.8_I_3_ QDs, the *R*_CT_ value was reduced, exhibiting promoted hole extraction and decreased energy loss on the back surface of MAPbI_3_. Furthermore, compared with the pristine and the CsSn_0.1_Pb_0.9_I_3_ QDs-added PSCs, the value of *C*_T_ increased, so that a shorter depletion width near the back surface of MAPbI_3_ (*W*_D_) could be deduced based on the following formulas, suggesting facilitated hole transfer.

4 $$C_{{\text{T}}} = \frac{{C_{1} C_{2} }}{{C_{1} + C_{2} }}$$

5 $$C_{1} = \frac{{\varepsilon_{{{\text{MAPbI}}_{{3}} }} A}}{{W_{{\text{D}}} }}$$

6 $$C_{2} = \frac{{\varepsilon_{{{\text{QD}}}} A}}{{d_{{{\text{QD}}}} }}$$

where *A* is the active area and *d*_QD_ is the width of the PQD layer. It is worth noting that the contact between MAPbI_3_ and the PQDs would form a hole depletion region in MAPbI_3_. Then, the contact between the PQDs and the carbon electrode would generate a Schottky barrier, which led to a hole depletion region in the PQD layer. Both the depletion regions in MAPbI_3_ and the PQDs contributed to the barrier capacitance value. For the PSC in the presence of CsSn_0.3_Pb_0.7_I_3_ QDs, the lower VB edge of the PQDs allowed more holes to migrate from the PQD layer to the MAPbI_3_ film. These holes gradually moved away from the MAPbI_3_/PQDs interface under the isotype heterojunction electric field, thereby increasing the *W*_D_. This might be the reason for the low *C*_T_ value of CsSn_0.3_Pb_0.7_I_3_ QDs-added device.

To get insight on the carrier transfer process, the steady-state PL spectra for the MAPbI_3_ films with and without tin-doped PQDs were measured. As shown in Fig. 7d, the PL peak intensity at about 775 nm was obviously decreased after the incorporation of CsSn_0.1_Pb_0.9_I_3_ QDs or CsSn_0.2_Pb_0.8_I_3_ QDs. There are two explanations for the weakening of PL intensity: First, the PQDs cause additional non-radiative pathways to capture photogenerated carriers; second, the higher VB edge of PQDs allows more photogenerated holes to migrate to the PQD layer; thus, the number of carriers participating in direct recombination is reduced. However, after adding CsSn_0.3_Pb_0.7_I_3_ QDs with more orthorhombic by-products and lower VB edge, the PL intensity increased, which showed that more carriers were limited in the perovskite film without being trapped by defects. Therefore, the PL quenching of the perovskite film with CsSn_0.1_Pb_0.9_I_3_ QDs or CsSn_0.2_Pb_0.8_I_3_ QDs was caused by the optimized band alignment promoting the hole extraction, instead of interfacial trap-assisted recombination.

**Fig. 8:**
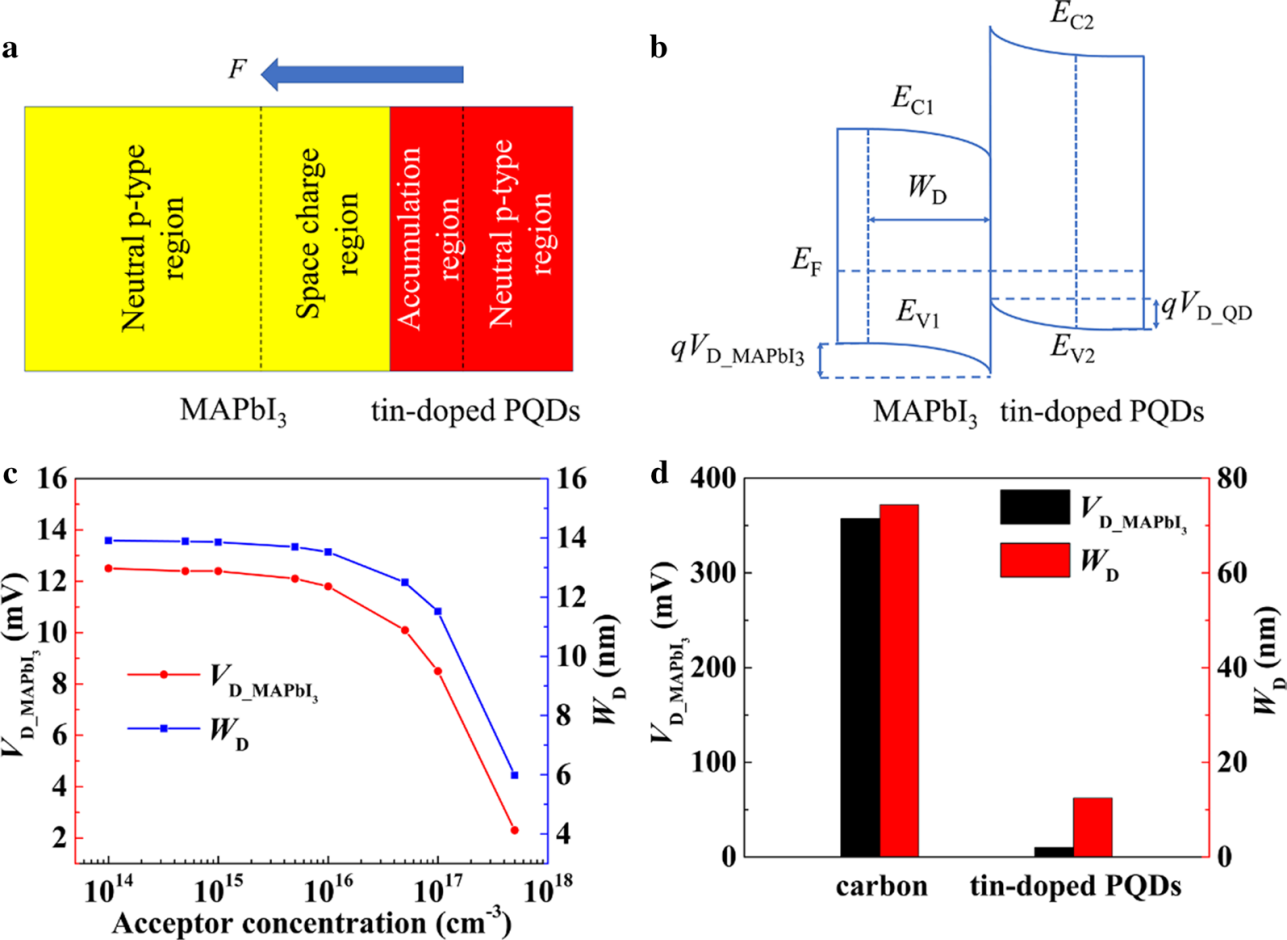
**a** The one-dimensional MAPbI_3_/PQDs heterojunction model. **b** Corresponding energy band diagram under the equilibrium condition. **c** and **d** Simulation results for MAPbI_3_/PQDs and MAPbI_3_/carbon heterojunctions

In order to further understand the effects of tin-doped PQDs on the hole transport in MAPbI_3_ films, a one-dimensional MAPbI_3_/tin-doped PQDs heterojunction model was constructed, shown in Fig. 8a. To simplify the analysis, this structure was regarded as a mutant isotype heterojunction, and MAPbI_3_ and tin-doped PQDs were determined to be p-type semiconductors. Theoretically, MAPbI_3_ is a kind of intrinsic semiconductor with low doping concentration. However, in the carbon-based perovskite PSCs with no HTLs, the perovskite layer needs to undergo p-type doping treatment. A small amount of DMSO was added in the precursor of perovskite to form a complex with PbI_2_, so that there were Pb vacancies in the perovskite, which made the perovskite become a p-type semiconductor. Moreover, Laban and Etgar utilized Mott–Schottky analysis to find that the acceptor concentration of MAPbI_3_ was 2.14 × 10^17^ cm^−3^, belonging to the doping level of p-type materials [38]. The contact of two semiconductors with different Fermi levels would form an electric field from the one with a high Fermi level to the another one with a low Fermi level. Consequently, the p–p isotype heterojunction energy band diagram under the equilibrium condition could be obtained, shown in Fig. 8b. According to the Poisson’s equation, the field continuity condition and the depletion approximation [39], barrier distributions of the isotype heterojunction were expressed by the following equations:

7 $$\exp \left( {\frac{{qV_{{{\text{D\_QD}}}} }}{{k_{{\text{B}}} T}}} \right) - \frac{{qV_{{{\text{D\_QD}}}} }}{{k_{{\text{B}}} T}} - 1 = \frac{{\varepsilon_{{{\text{MAPbI}}_{{3}} }} N_{{{\text{A\_MAPbI}}_{{3}} }} }}{{\varepsilon_{{{\text{QD}}}} N_{{{\text{A\_QD}}}} }}\frac{{qV_{{{\text{D\_MAPbI}}_{{3}} }} }}{{k_{{\text{B}}} T}}$$

8 $$qV_{{{\text{D\_MAPbI}}_{{3}} }} + qV_{{{\text{D\_QD}}}} = E_{{{\text{Fermi\_QD}}}} - E_{{{\text{Fermi\_MAPbI}}_{{3}} }}$$

9 $$E_{{{\text{Fermi}}}}^{{\text{p}}} = \frac{1}{2}\left( {E_{{{\text{CB}}}} + E_{{{\text{VB}}}} } \right) - \frac{1}{2}k_{{\text{B}}} T\ln \left( {\frac{{N_{{\text{C}}} }}{{N_{{\text{V}}} }}} \right) - k_{{\text{B}}} T\ln \left( {\frac{{N_{{\text{a}}} }}{{n_{{\text{i}}} }}} \right)$$

10 $$W_{{\text{D}}} = \sqrt {\frac{{2\varepsilon_{{{\text{MAPbI}}_{{3}} }} V_{{{\text{D\_MAPbI}}_{{3}} }} }}{{qN_{{{\text{A\_MAPbI}}_{{3}} }} }}}$$

where *q* is the elementary charge and *ε*_QD_ and *N*_A_QD_ are the dielectric coefficient and the acceptor concentration for tin-doped PQDs, respectively. *V*_D_MAPbI3_ and *V*_D_QD_ are the potential difference in MAPbI_3_ and tin-doped PQDs in turn. *E*_Fermi_MAPbI3_ and *E*_Fermi_QD_ stand for the Fermi levels of MAPbI_3_ and tin-doped PQDs, respectively. *k*_B_ is the Boltzmann constant and *T* is the room temperature. *N*_C_ and *N*_V_ are the effective density of states of electrons in conduction band and the effective density of states of holes in valence band, respectively. *N*_a_ is the acceptor concentration, *n*_i_ is the intrinsic carrier concentration and *W*_D_ is the depletion width in MAPbI_3_. The simulation results are exhibited in Fig. 8c. As the acceptor concentration of tin-doped PQDs increased, both *V*_D_MAPbI3_ and *W*_D_ showed downward trends, indicating that the hole transfer process in the MAPbI_3_ film was gradually facilitated. Besides, less electrons would be drifted to the interface between MAPbI_3_ and the PQD layer to recombine with holes. On the contrary, the direct contact between MAPbI_3_ and the carbon electrode would generate a large Schottky barrier in MAPbI_3_, resulting in higher values of *V*_D_MAPbI3_ and *W*_D_, shown in Fig. 8d. In one word, MAPbI_3_ films modified by tin-doped PQDs with higher acceptor concentrations would be provided with much enhanced hole transport performance. This simulation result explained why the photovoltaic performance of the CsSn_0.2_Pb_0.8_I_3_ QDs-added PSC was better than the pristine and the CsSn_0.1_Pb_0.9_I_3_ QDs-added devices.

## Conclusions

In summary, tin-doped PQDs were added between MAPbI_3_ and the carbon electrode for enhanced PSC performance, due to their flexible energy levels and self-p-type doping effects. Particularly, with the incorporation of CsSn_0.2_Pb_0.8_I_3_ QDs, the PCE value could be improved from 12.80 to 14.22%, in comparison with the pristine device. It was attributed to the band alignment and the appropriate Sn^2+^ doping content of the PQDs facilitating the hole extraction. This work is prospected to provide a direction for the interface optimization of carbon-based PSCs based on PQDs.

## Supplementary Information

**Additional file 1: Fig. S1**. EDS mappings of different tin-doped PQDs under HAADF STEM patterns. **a** CsSn_0.1_Pb_0.9_I_3_ QDs, **b** CsSn_0.2_Pb_0.8_I_3_ QDs, c CsSn_0.3_Pb_0.7_I_3_ QDs. **Fig. S2**. UPS data and Tauc plots for **a** CsSn_0.1_Pb_0.9_I_3_ QDs, **b** CsSn_0.2_Pb_0.8_I_3_ QDs, **c** CsSn_0.3_Pb_0.7_I_3_ QDs and **d** MAPbI_3_. The valence band (VB) edges were calculated by E_VB_ = − (21.21 eV − E_Cut-off_ + E_Low-binding_). Then, the conduction band (CB) edges were calculated by E_CB_ = E_VB_ + E_g_. **Fig. S3**. XPS measurement on the film with the structure of FTO/c-TiO_2_/m-TiO_2_/MAPbI_3_/PQDs. **Fig. S4**. Bar charts indicating photovoltaic parameters of ten CsSn_0.2_Pb_0.8_I_3_ QDs-added devices at the PQD concentration of 10 mg mL^−1^. **Fig. S5**. Error bars of photovoltaic parameters for different PSCs. **Fig. S6**. Normalized PCE values for CsSn_0.2_Pb_0.8_I_3_ QDs-added and the pristine devices at 60% humidity in room temperature. **Table S1**. EDS chemical composition analysis for CsSn_0.1_Pb_0.9_I_3_ QDs. **Table S2**. EDS chemical composition analysis for CsSn_0.2_Pb_0.8_I_3_ QDs. **Table S3**. EDS chemical composition analysis for CsSn_0.3_Pb_0.7_I_3_ QDs. **Table S4**. Photovoltaic parameters of different PSCs. **Table S5**. Photovoltaic parameters of CsSn_0.2_Pb_0.8_I_3_ QDs-added PSCs under different concentrations of the PQD solution.

## Data Availability

The datasets used and/or analyzed during the current study are available from the corresponding author on reasonable request.

## Abbreviations

PSC(s): Perovskite solar cell(s)
PQD(s): Perovskite quantum dot(s)
QD(s): Quantum dot(s)
HTL(s): Hole-transporting layer(s)
PCE: Power conversion efficiency
ODE: 1-Octadecene
OA: Oleic acid
OAM: Oleylamine
MeOAc: Methyl acetate
TOP: Trioctylphosphine
MAI: Methylammonium
DMSO: Dimethylsulfoxide
DMF: *N*,*N*-Dimethylformamide
FTO: Fluorine-doped SnO_2_
c-TiO_2_: Compact TiO_2_
m-TiO_2_: Mesoporous TiO_2_
TEM: Transmission electron microscope
SAED: Selected area electron diffraction
EDS: Energy dispersive X-ray spectroscopy
VB: Valence band
PL: Photoluminescence
IPCE: Incident photon-to-electron conversion
EIS: Electrochemical impedance spectroscopy
XRD: X-ray diffraction
