# Supplementary material for: Effects of CsSnxPb1−xI3 Quantum Dots as Interfacial Layer on Photovoltaic Performance of Carbon-Based Perovskite Solar Cells
Source: Nanoscale Res Lett. 2021 Apr 29;16:74. doi: 10.1186/s11671-021-03533-y (PMC8085196; doi:10.1186/s11671-021-03533-y)
Supplement: Supplementary file 1 — Additional file 1: Fig. S1. EDS mappings of different tin-doped PQDs under HAADF STEM patterns. a CsSn0.1Pb0.9I3 QDs, b CsSn0.2Pb0.8I3 QDs, c CsSn0.3Pb0.7I3 QDs. Fig. S2. UPS data and Tauc plots for a CsSn0.1Pb0.9I3 QDs, b CsSn0.2Pb0.8I3 QDs, c CsSn0.3Pb0.7I3 QDs and d MAPbI3. The valence band (VB) edges were calculated by EVB = − (21.21 eV − ECut-off + ELow-binding). Then, the conduction band (CB) edges were calculated by ECB = EVB + Eg. Fig. S3. XPS measurement on the film with the structure of FTO/c-TiO2/m-TiO2/MAPbI3/PQDs. Fig. S4. Bar charts indicating photovoltaic parameters of ten CsSn0.2Pb0.8I3 QDs-added devices at the PQD concentration of 10 mg mL−1. Fig. S5. Error bars of photovoltaic parameters for different PSCs. Fig. S6. Normalized PCE values for CsSn0.2Pb0.8I3 QDs-added and the pristine devices at 60% humidity in room temperature. Table S1. EDS chemical composition analysis for CsSn0.1Pb0.9I3 QDs. Table S2. EDS chemical composition analysis for CsSn0.2Pb0.8I3 QDs. Table S3. EDS chemical composition analysis for CsSn0.3Pb0.7I3 QDs. Table S4. Photovoltaic parameters of different PSCs. Table S5. Photovoltaic parameters of CsSn0.2Pb0.8I3 QDs-added PSCs under different concentrations of the PQD solution. [file 11671_2021_3533_MOESM1_ESM.docx]

**Effects of CsSn*_x_*Pb_1-_*_x_*I_3_ Quantum Dots as Interfacial Layer on Photovoltaic Performance of Carbon-Based Perovskite Solar Cells**

Chi Zhang, Zhiyuan He, Xuanhui Luo, Rangwei Meng, Mengwei Chen, Haifei Lu and Yingping Yang*

*correspondence: [ypyang@whut.edu.cn](mailto:ypyang@whut.edu.cn)

Department of Physics, School of Science, Wuhan University of Technology, Wuhan 430070, P. R. China


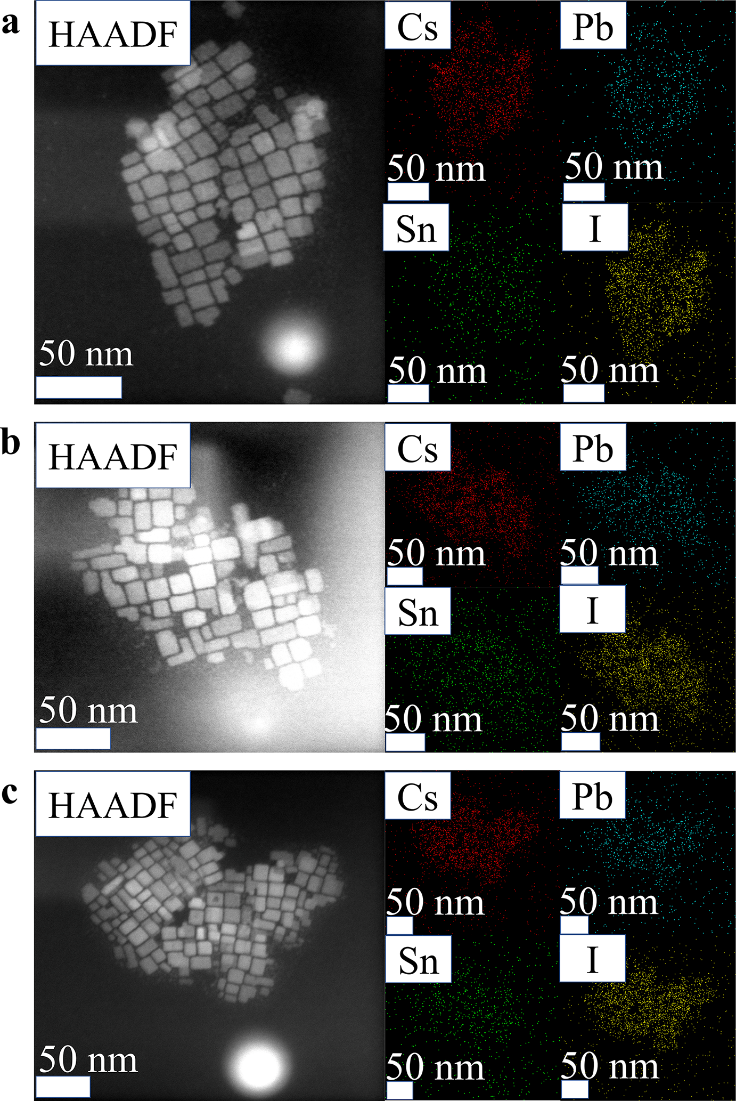


**Fig. S1** EDS mappings of different tin-doped PQDs under HAADF STEM patterns. **a** CsSn_0.1_Pb_0.9_I_3_ QDs, **b** CsSn_0.2_Pb_0.8_I_3_ QDs, **c** CsSn_0.3_Pb_0.7_I_3_ QDs.

**Table S1** EDS chemical composition analysis for CsSn_0.1_Pb_0.9_I_3_ QDs.

| Element | Atomic  fraction (%) | Atomic  error (%) | Mass  fraction (%) | Mass  error (%) | Fit  error (%) |
| --- | --- | --- | --- | --- | --- |
| Cs | 14.08 | 2.31 | 12.89 | 1.68 | 2.15 |
| Sn | 3.31 | 0.50 | 2.71 | 0.30 | 7.37 |
| Pb | 22.09 | 3.68 | 31.52 | 4.21 | 1.38 |
| I | 60.52 | 9.85 | 52.89 | 6.80 | 0.81 |

**Table S2** EDS chemical composition analysis for CsSn_0.2_Pb_0.8_I_3_ QDs

| Element | Atomic  fraction (%) | Atomic  error (%) | Mass  fraction (%) | Mass  error (%) | Fit  error (%) |
| --- | --- | --- | --- | --- | --- |
| Cs | 17.44 | 2.79 | 16.16 | 2.08 | 1.95 |
| Sn | 5.65 | 0.77 | 4.68 | 0.46 | 5.24 |
| Pb | 19.89 | 3.25 | 28.73 | 3.81 | 1.88 |
| I | 57.02 | 9.05 | 50.44 | 6.40 | 0.62 |

**Table S3** EDS chemical composition analysis for CsSn_0.3_Pb_0.7_I_3_ QDs.

| Element | Atomic  fraction (%) | Atomic  error (%) | Mass  fraction (%) | Mass  error (%) | Fit  error (%) |
| --- | --- | --- | --- | --- | --- |
| Cs | 17.37 | 2.79 | 16.47 | 2.12 | 2.09 |
| Sn | 7.72 | 1.02 | 6.54 | 0.59 | 3.65 |
| Pb | 15.98 | 2.63 | 23.62 | 3.15 | 2.28 |
| I | 58.94 | 9.40 | 53.37 | 6.79 | 0.86 |


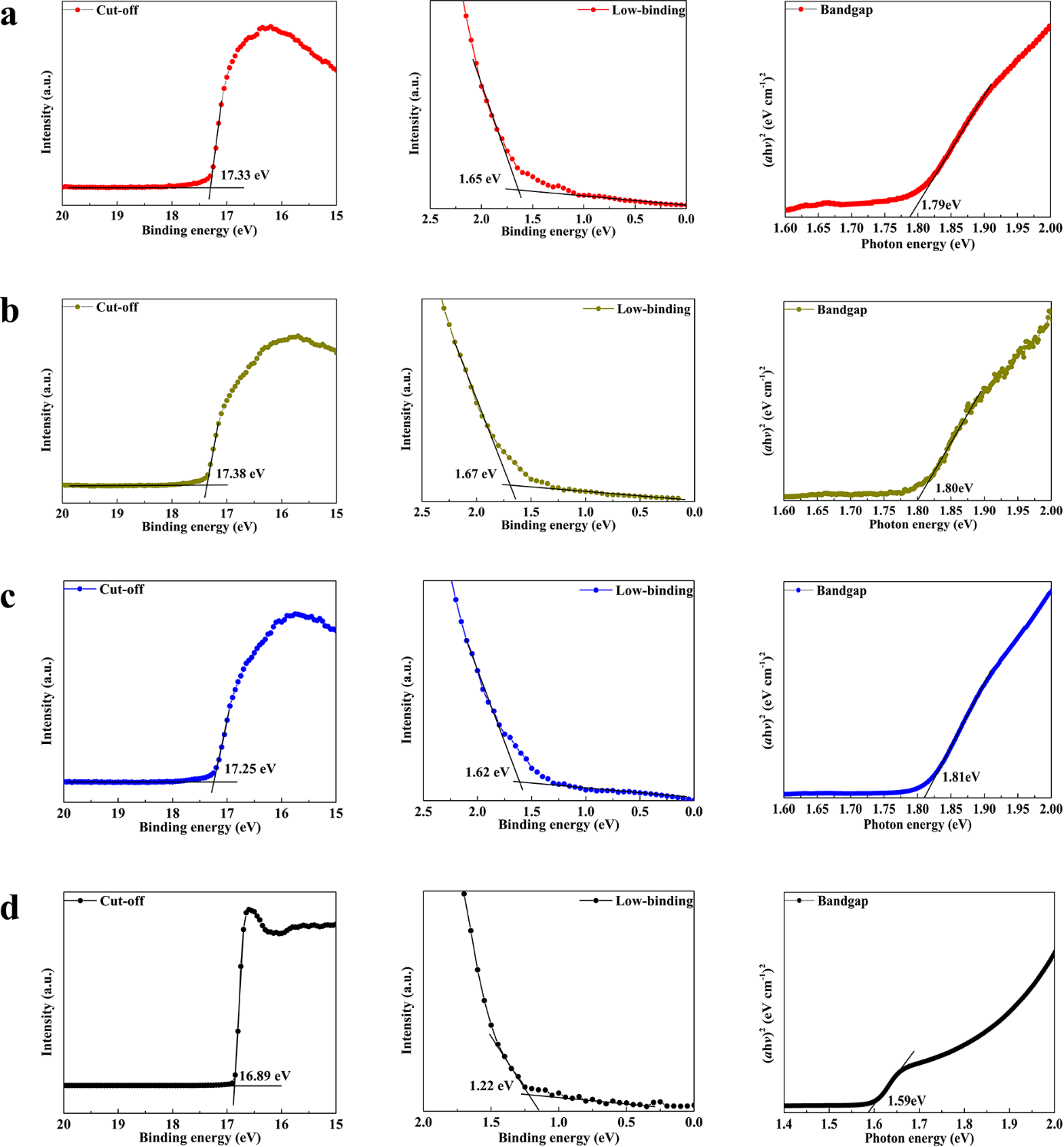


**Fig. S2** UPS data and Tauc plots for **a** CsSn_0.1_Pb_0.9_I_3_ QDs, **b** CsSn_0.2_Pb_0.8_I_3_ QDs, **c** CsSn_0.3_Pb_0.7_I_3_ QDs and **d** MAPbI_3_. The valence band (VB) edges were calculated by E_VB_ = − (21.21 eV − E_Cut-off_ + E_Low-binding_). Then, the conduction band (CB) edges were calculated by E_CB_ = E_VB_ + E_g_.


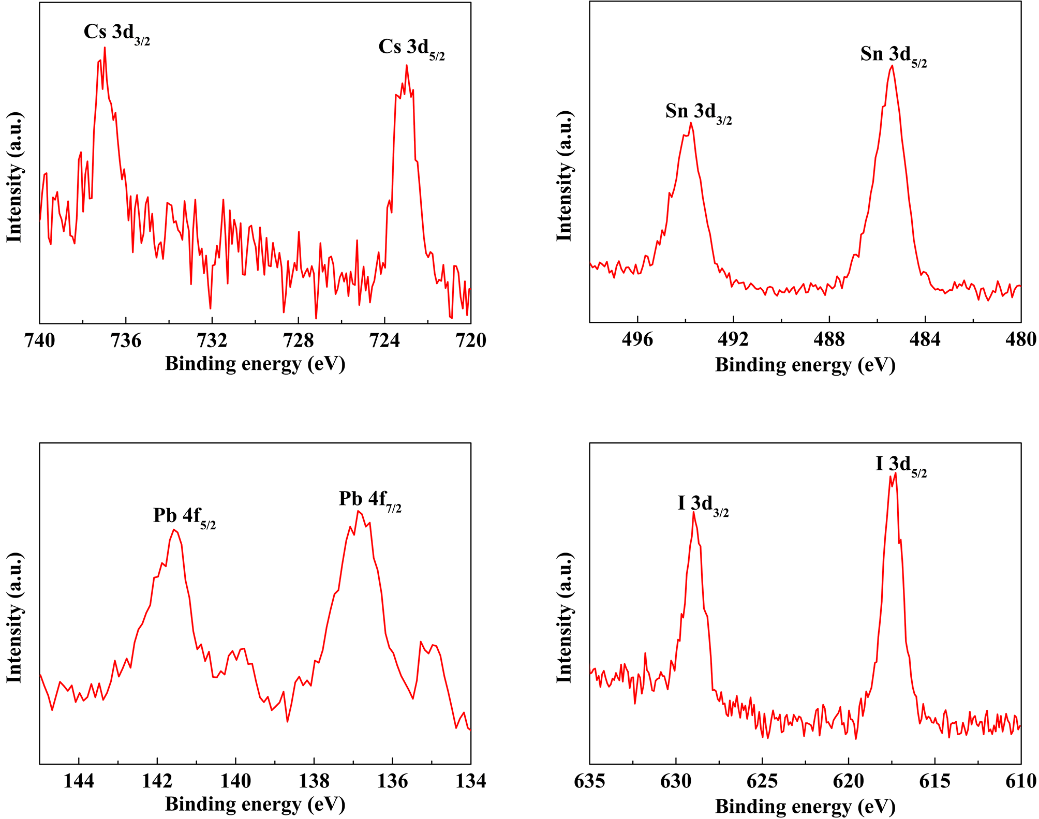


**Fig. S3** XPS measurement on the film with the structure of FTO/c-TiO_2_/m-TiO_2_/MAPbI_3_/PQDs.

**Table S4** Photovoltaic parameters of different PSCs

| Device | *V*_oc_ (V) | *J*_sc_ (mA cm^-2^) | FF (%) | PCE (%) |
| --- | --- | --- | --- | --- |
| Control | 0.98 | 22.58 ± 0.11 | 55.44 ± 1.14 | 12.29 ± 0.09 |
| With CsSn_0.1_Pb_0.9_I_3_ QDs | 1.01 | 23.28 ± 0.13 | 54.96 ± 3.63 | 12.91 ± 0.13 |
| With CsSn_0.2_Pb_0.8_I_3_ QDs | 1.04 | 23.46 ± 0.15 | 55.86 ± 1.48 | 13.58 ± 0.16 |
| With CsSn_0.3_Pb_0.7_I_3_ QDs | 0.96 | 14.80 ± 3.22 | 45.63 ± 11.53 | 6.48 ± 0.82 |

**Table S5** Photovoltaic parameters of CsSn_0.2_Pb_0.8_I_3_ QDs-added PSCs under different concentrations of the PQD solution.

| Concentration (mg mL^-1^) | *V*_oc_ (V) | *J*_sc_ (mA cm^-2^) | FF (%) | PCE (%) |
| --- | --- | --- | --- | --- |
| 5 | 1.00 | 23.58 ± 0.15 | 56.28 ± 2.75 | 13.29 ± 0.25 |
| 10 | 1.04 | 23.46 ± 0.15 | 55.86 ± 1.48 | 13.58 ± 0.16 |
| 15 | 1.01 | 22.86 ± 0.78 | 49.25 ± 6.37 | 11.38 ± 0.73 |
| 20 | 0.99 | 22.48 ± 0.21 | 49.06 ± 2.60 | 10.97 ± 0.20 |

The variance values of the open circuit voltage of different devices were so small that could be negligible.





**Fig. S4** Bar charts indicating photovoltaic parameters of ten CsSn_0.2_Pb_0.8_I_3_ QDs-added devices at the PQD concentration of 10 mg mL^-1^.





**Fig. S5** Error bars of photovoltaic parameters for different PSCs.





**Fig. S6** Normalized PCE values for CsSn_0.2_Pb_0.8_I_3_ QDs-added and the pristine devices at 60% humidity in room temperature.
